# Winanga-Li (I Hear You): Privileging Voices and Experiences of Aboriginal Parents’ Journey with Their Gaaynggal (Baby) Through a Neonatal Intensive Care Unit

**DOI:** 10.3390/ijerph22040554

**Published:** 2025-04-03

**Authors:** Jessica Bennett, Jamie Bryant, Kade Booth, Michelle Kennedy

**Affiliations:** 1 School of Medicine and Public Health, The University of Newcastle, Callaghan, NSW 2308, Australia; jamie.bryant@newcastle.edu.au (J.B.); k.booth@newcastle.edu.au (K.B.); michelle.kennedy11@newcastle.edu.au (M.K.); 2 John Hunter Children’s Hospital, Hunter New England Health, New Lambton Heights, NSW 2305, Australia; 3 Hunter Medical Research Institute, The University of Newcastle, Callaghan, NSW 2308, Australia

**Keywords:** Aboriginal and Torres Strait Islander health, indigenous health, neonatal care, cultural safety

## Abstract

Aboriginal parents experience neonatal intensive care settings at a higher rate than non-Indigenous parents. We sought to explore Aboriginal parents’ experiences of having a gaaynggal (baby) admitted to a neonatal intensive care unit (NICU) in order to improve culturally safe neonatal care environments. The yarning method was used to collect the qualitative data of 15 Aboriginal parents’ stories. Thematic analysis and collaborative yarning were used to determine themes. The themes emerging from the stories included Trauma and its triggers in the NICU; Aboriginal cultural caring practices are not upheld in the NICU; Covert racism and biases impact culturally safe experiences; Health provider communication can obstruct parents’ experience of cultural safety; and Recommendations to uphold culturally safe care in NICU. Culturally safe care practices have been identified as needed, to improve cultural safety in neonatal settings. Through further education and training, the facilitation of Aboriginal family connections and support groups, culturally inclusive spaces for parents and their kinship systems, and increasing Aboriginal staff representations across all levels of health professional experience, cultural safety for Aboriginal parents and gaaynggal can be increased.

## 1. Introduction

Past and present coloniality in Australia has disrupted cultural caring practices of Aboriginal gaayli (children), including the disruption of kinship systems, mother crafting, and community ways of parenting the next generation of Aboriginal gaayli [1,2]. The rates of admissions to a neonatal intensive care unit (NICU) for Aboriginal and Torres Strait Islander gaaynggal (babies) remain significantly higher than for non-Aboriginal infants (24.5% vs. 16.6%), with no change in trends over time [3]. The primary reasons for admission to a NICU for Aboriginal gaaynggal are prematurity, respiratory distress, and jaundice [4]. Prematurity is the leading cause of high mortality rates [5], and Aboriginal and Torres Strait Islander gaaynggal experience neonatal deaths (deaths within 28 days of birth) 1.8 times more often than non-Aboriginal infants [3]. While over-representation in the NICU is evident, research and policy efforts to date have focused on prevention of poor neonatal outcomes, such as decreasing low-birth-weight births [6]. While critical, this does not address the present experiences of Aboriginal and Torres Strait Islander families who experience a neonatal setting with poor neonatal outcomes, and it does not demonstrate the need for culturally safe care.

Current research in the neonatal setting has reported that an admission is both a traumatic and stressful experience in the parenting journey [7]. Parents have the potential to be impacted by experiences of grief, isolation, anxiety, and exhaustion [8]. Throughout the literature, for non-Indigenous experiences, it has been reported that an admission to a NICU disrupts the normal transition to parenthood and bonding processes [9]. These disruptions have been reported to add additional complexities to the parenting role as they navigate both the emotional issues of having an unwell gaaynggal and the physical separation [9]. Experiences such as environmental stressors, as explained by parents, have been documented to effect attachment and breastfeeding and have impacted travel and financial barriers and the meeting of the needs of their other children [7]. It is evident that by increasing levels of parental involvement with their gaaynggal in the NICU, parental experiences are improved, and poor infant outcomes and environmental stressors are decreased [10]. It is likely that for Aboriginal gaaynggal, these experiences are heightened when compounded with the disruption and harms caused by colonisation in Australia.

Recent reports from Indigenous First Nations peoples in Canada and Māori people in New Zealand exploring parents’ experiences offer the need for Indigenous specific trauma-informed care, culturally safe care coordination, family-centred environments, and better communication [11,12,13]. This is recommended to be embedded through an Indigenous lens [14]. While there is some movement globally to represent Indigenous parents’ experiences in the NICU, there is no research on Aboriginal parents’ experiences in Australia. This study prioritises the voices of Aboriginal parents to make meaningful change, and to embed culturally safe neonatal care. To our knowledge, this is the first paper that details the experiences of Aboriginal parents having their gaaynggal admitted to a NICU.

This study was developed in response to community priorities. As an Aboriginal woman, mother, and neonatal care nurse, I aimed to winanga-li (to hear) and privilege the voices of Aboriginal parents. This study seeks to uphold the definition of cultural safety and aims to explore Aboriginal parents’ experiences of having a gaaynggal admitted to a neonatal intensive care unit in one of New South Wales’s biggest NICUs, John Hunter Children’s Hospital. This study is being conducted to improve culturally safe care by informing and providing recommendations for evidence-based clinical practice in culturally safe care for Aboriginal and Torres Strait Islander gaaynggal and families in neonatal settings in Australia.

## 2. Materials and Methods

Winanga-li in Gamilaraay means ‘I hear you’. This study sought to listen and hear Aboriginal parents’ stories using the yarning method [15]. The yarning method is an Indigenous method of qualitative data collection [15] that privileges Indigenous ways of knowing, being, and doing [16]. This study used three types of yarning: social yarning, research topic yarning, and therapeutic yarning [15]. Social yarning takes place at the beginning of the interaction between the Aboriginal researcher and Aboriginal parent and focuses on building trust and connection. Once trust is established, research topic yarning is introduced to provide the opportunity to discuss the research in detail, ask questions about the topic, and understand what their role and involvement will include. Therapeutic yarning is the process of conducting the interview itself, where the researcher is positioned to listen to the parent’s experience and story. This is the only part of the yarning process that is audio-recorded and analysed. The yarning method was applied to uphold Indigenous ways of knowledge sharing [15], allowing parents to share their NICU experiences from their own perspectives. The yarning took place during the parents’ gaaynggal’s admission or up to 3 months post-discharge.

### 2.1. Research Team

Conducting culturally safe and responsive research with Aboriginal parents within a NICU environment requires relational research practices. Relationality, drawing on our Indigenous standpoint, was integral to the research practice [17] and provided the opportunity to challenge current deficits in the discourses on Aboriginal health, as the researcher is able to relate to the lived experience [14]. This study was conceptualized, led, and conducted by a Gamilaroi woman, a PhD student and neonatal registered nurse, JBe, as part of doctoral research. This is a unique position for both the research*er* and the research*ed* throughout this process [18]. The recruited hospital has been JBe’s workplace for over 10 years. As a mother, she also experienced high-risk pregnancy and medical care, and NICU admissions with her own gaaynggal. JBe was supported by MK, a Wiradjuri woman, mother, and Aboriginal health researcher with experience of the NICU as a parent, alongside non-Indigenous researchers JBr and KB, who have extensive experience in Aboriginal and Torres Strait Islander health research and qualitative methodologies. Aboriginal and Torres Strait Islander community researchers, who work alongside the research team, are integral to the research partnership. They bring their knowledge and expertise from a range of diverse settings, including academic and research institutions and public health services, including clinical settings. We acknowledge that our standpoint, world view, and experiences have shaped the design, implementation, analysis, and interpretation process.

### 2.2. Ethics and Governance

This study was conducted and reported in accordance with the international ‘CONSIDER’ [19] statement and the National Health and Medical Research Council’s Guidelines for ethical conduct in Aboriginal and Torres Strait Islander health research [20] and the Aboriginal Health and Medical Research Council’s AH&MRC ethical guidelines: key principles (2020) V2.0 [21]. Ethics approval was provided by the Human Research Ethics Committee (HREC) of AH&MRC of New South Wales, Australia (AH&MRC 2080/23), the Hunter New England Local Health District HREC (2023/ETH00910), and the University of Newcastle HREC (H-2023-0165). The standard of reporting qualitative research (SRQR) has also been used to guide the reporting of study findings [22].

The research design, implementation, analysis, interpretation, and dissemination were guided by an Aboriginal Governance Committee. The committee consisted of Aboriginal and Torres Strait Islander nurses, doctors, midwives, an Aboriginal liaison officer, and researchers in the local area, who are knowledge holders in this space.

### 2.3. Participants and Recruitment

Parents were eligible if they self-identified as Aboriginal and/or Torres Strait Islander women, men, and/or non-binary persons. Parents were to be aged 16 years and over and to have had a gaaynggal admitted to the John Hunter Children’s Hospital (JHCH) NICU at any gestational age or up to 3 months post-discharge from the neonatal service. To privilege Aboriginal experiences, non-Aboriginal and/or Torres Strait Islander people were excluded from this study. No Torres Strait Islander parents were admitted during the recruitment period; therefore, Aboriginal would be used throughout. Parents were recruited face-to-face in the NICU by JBe. The NICU staff notified JBe if a parent self-identified as Aboriginal. Recruitment and data collection occurred while JBe was on leave from her nursing role at the hospital to ensure there was no conflict of interest and that participants felt comfortable discussing their experiences without concern for their interactions with hospital staff.

JBe approached parents in person to introduce herself and commence social and research topic yarning [15] and gauge interest in participating in the study. JBe used her discretion as a health professional to determine whether it was appropriate to conduct social yarns with the parents. To ensure that the health and wellbeing of the gaaynggal and parents were prioritized, parents who were distressed or for whom it was otherwise not appropriate to discuss participation were not approached. Social yarning started at the bedside; JBe then established a rapport while also informally checking in on the wellbeing of the parents. With the goal of creating a safe and trusting environment, JBe opened with her Indigenous standpoint to position herself as an Aboriginal woman, a mother, and a nurse [23]. If the parents were interested in participating, research topic yarning commenced, providing an opportunity to offer context to the research and to explain why it was being conducted, as well as to answer any questions the parents had prior to providing informed consent. Information sheets were then provided at the bedside. If the parents wanted to participate in the study, an interview time was scheduled for a time that was most convenient for the parents. This occurred either on the day of recruitment or up to 3 weeks post-yarning, depending on the availability and preferences of the parents.

### 2.4. Data Collection

Interviews were conducted between late August 2023 and mid-May 2024. The interviews were conducted face-to-face (13 parents) or over the phone (2 parents). Face-to-face interviews were held in a family room within the NICU, as it was in close proximity and the parents were able to get back to their gaaynggal when needed. The interviews were between 22 and 80 min (an average of 36.29 min). Before data collection commenced, social yarning and research yarning occurred again to reestablish connection and obtain informed consent for participation. Therapeutic yarning would then only occur after ascertaining that the gaaynggal was medically stable, and if it was suitable to conduct the interview with the parents in their current stage of their NICU journey. If the timing of the interview was deemed inappropriate based on the gaaynggal’s medical condition or the parents’ discretion, JBe would reschedule the interview and would offer and provide cultural support.

Most yarns commenced organically, others began with the question, “tell me a bit about your birthing experience?”. During the interview, reciprocal yarning became common between the parents and researcher, due to the researcher’s relationality, as many of the parents had questions in relation to their gaaynggal, their care, and cultural support. This also happened after the interview, when JBe would go back and support parents at the bedside, if the parents asked for additional cultural support during the interview. In line with the aims of the study, the flow of yarns was guided by the participants to allow them to share their stories and experiences. At times, JBe would offer prompting questions, to clarify aspects of the stories as the conversation progressed. The interviews did not always follow the same trajectory.

Basic demographics were collected from the parents to describe the circumstances of their gaaynggal’s admission. The Australian Statistical Geography Standard (ASGS) Edition 3 [24] was used to determine a classification based on parents’ location of residence, to contextualize their experience. The yarning interviews were audio-recorded using the in-built recording option on a university-owned iPad. The audio-recorded interviews were transcribed verbatim using a professional transcription service. The transcriptions were checked for consistency by JBe and sent to the participants who indicated a preference to receive a copy. Three participants received a copy of the transcript of their stories to make any necessary edits. No revisions were requested. At the end of the interview, the parents received a Coles or Woolworths voucher worth AUD 50 for participating.

### 2.5. Analysis

Initial data analysis was conducted using NVivo 14 software by the first author; JBe conducted a thematic analysis following the steps described by Braun and Clarke [25]. JBe independently read and re-read interviews to familiarize themselves with the data and coded the stories line-by-line. The data were then dual coded for the initial 6 stories (JBen and KB) to provide the opportunity for the sharing of interpretations between the researchers and to include a collaborative approach to the coding process. The remaining data were then coded by JBen. A codebook was developed, despite not typically being suggested by Braun and Clarke [25] to create clear summaries and definitions, as a tool to ignite robust discussion with the research team and the Aboriginal Governance group during the collaborative analysis approach used within this study.

Additional analysis was then conducted in a workshop model with Aboriginal and Torres Strait Islander community researchers from the research team and the Aboriginal Governance group to inform analysis through collaborative yarning. This is in alignment with standard practice and allows the privileging of the knowledge holders and their expertise, while also continually prioritizing Indigenous methods in conducting meaningful and beneficial research [26]. During the workshop, attendees were provided with a copy of the full analysis, including all quotations from the parents and the broad themes. Ongoing collaborative yarning was used to check consensus about themes and interpretations. Notes were collected by JBe throughout the session and collated to re-define the themes and interpretations that were generated. JBe presented the updated findings to the research team in person and the wider governance group by email before commencing further research dissemination.

## 3. Results

This study privileged the voices and experiences of 12 Aboriginal mothers and 3 Aboriginal fathers (13 gaaynggal with one set of twins). All the parents identified as Aboriginal with connections to six different Aboriginal nations in the surrounding areas: Awabakal, Aniwan, Worimi, Wonnarua, Gamilaroi, and Birripi. No self-identifying Torres Strait Islander people were admitted during the recruitment period. No yarns were stopped by the parents during the interview. All the mothers and fathers approached to participate in the study were given an information sheet and an opportunity to ask questions during the research topic yarning and had communicated verbal interest. The parents were then followed up by JBe to see if the parents would like to organise an interview time. Nine parents could not be followed up due to transfers back to hospitals closer to their homes or because they were discharged. No parents declined the study.

The demographics of the gaaynggal are presented in Table 1. The gaaynggal’s gestational ages were between 26 weeks and 3 days gestational age and 40 weeks and 4 days gestational age. Most of the gaaynggal experienced both intensive care and special care as medical treatment during their admission. Most of the gaaynggal were from regional areas, which meant most were transferred “off country” either in utero (before birth) or ex utero (after birth). Off country refers to the disconnection from an Aboriginal person’s ancestorial homelands, community connection, and kinship systems, due to having to travel away from their home to receive medical care on country that is not their own [27].

Five themes were drawn from these stories and reflect findings that have implications for clinical practice: Theme 1: Trauma and its triggers in the NICU; Theme 2: Aboriginal cultural caring practices are not upheld in the NICU; Theme 3: Covert racism and biases impact culturally safe experiences; Theme 4: Health provider communication can obstruct parents’ experience of cultural safety; and Theme 5: Recommendations to uphold culturally safe care in NICU.

**Theme 1: Trauma and its triggers in the NICU**

The parents described the ways in which the NICU environment and experiences can trigger trauma responses. Most parents raised concerns about their gaaynggal’s survival, potential for lifelong disability, and physical and emotional wellbeing. These concerns were heightened by the fact that the NICU was not a topic discussed within the community, leaving them uncertain and unprepared for the experience. Few parents knew of the NICU due to a member of the immediate family having a gaaynggal admitted. Some parents expressed that this was triggering pre-existing trauma, especially that associated with child removal and fears that their gaaynggal was being taken away long term. *“I freaked out. I thought they were stealing him. Why are you taking my child way? What have I done? No one actually explained that we weren’t going to be in the same room. And as soon as they walked him the opposite way, I’m like, why are you taking my baby away from me?”* (Mother 9—Gamilaroi).

Experiences of separation had a negative impact on most of the parents. Several families experienced separation by geographical difference (transfer of a gaaynggal by trained neonatal retrieval teams from a rural hospital that was unable to provide high-acuity medical care). Parents felt that they were not able to uphold their rights to cultural parenting practices due to the disruption to kinship caring systems, and there were the challenges presented by transferral from rural hospitals, where parents were separated and far from their cultural and family support systems. For example, one father was left in a neighbouring town with no resources and no money and was unable to support his family as he was unable to be transferred with the mother and gaaynggal. “*Like there’s only so much you can do for us, but when it comes to complications with babies, they should have their fathers around.”* (Father 3—Aniwan)

Significant travel from ‘country’ (homelands) and being in a city that caused feelings of anxiousness due to the new surroundings had the parents feeling unprepared and unsupported and not able to parent the way they needed to. This added to the trauma of having an unwell or premature gaaynggal. A mother of a 26.4-week-old gaaynggal experienced this for over 13 weeks; she said, “*Especially from most Aboriginal communities, they’re remote or inland. Where this is a city, so I’ve never seen so many buses in my life for the same number. It is ridiculous. Whereas we only have five buses back where we’re from and they go to specific places.”* (Mother 15—Gamilaroi).

**Theme 2: Aboriginal cultural caring practices are not upheld in the NICU**

Parents reported that caring for our gaaynggal is culturally significant to our ways of knowing, being, and doing. Over half of the parents reported that there were barriers in their opportunity to uphold cultural ways of parenting in the NICU. Parents frequently expressed that they wanted to feel included and not be bystanders in the care being provided to their gaaynggal. This included having a family-centred approach, not being dismissed or overridden by staff, and having an ability to advocate or consent on their gaaynggal’s behalf, which was reflected as essential. Parents declared that it was their right to receive appropriate communication about their gaaynggal’s health and wellbeing. The need for improved communication did not vary between parents, despite the acuity or length of stay of the gaaynggal. “*We just would like to be included more as parents and Aboriginals*.” (Mother 2—Aniwan).

Parents expressed that cultural caring extends beyond the westernized constraints of parenting and includes kin and community. The inability to have extended family and community support within the NICU environment due to hospital rules and restrictions left parents frustrated. In addition, there was a lack of understanding of the Aboriginal father’s role in parenting and personal experience in raising other gaayli. These experiences extended to key nurturing practices, including breastfeeding, where many mothers where left feeling like they were ‘*failing*’ when staff tried to provide breastfeeding techniques. This was felt by parents who had experienced both short-term and long-term stays. Some of the parents expressed that breastfeeding was a practice that could be learnt culturally, through aunties, mothers, and sisters in the family. As one mother described, “*They put baby in a position and that, I know what position my baby’s got to be in, how they’ve got to lay and that.”* (Mother 10—Kamilaroi).

Breastfeeding was seen as a strength by mothers as they were able to provide for their *gaaynggal,* but it was experienced as a barrier in the NICU environment when required to pump and supply for their gaaynggal, which was unnatural to breastfeeding itself.

Several parents reported a discovery of inherent strength as Aboriginal people during their NICU admission and how it differs to western views of health. One father spoke of the resilience and strength our gaaynggal have, due to our ongoing connection to culture and how our health and wellbeing are stronger due to that connection as Aboriginal people. “*But black people, we have different immune systems and probably stronger immune systems, but they don’t see that.*” (Father 3—Aniwan).

Many of the mothers felt that it was crucial to try to remain positive during this time, as they knew their gaaynggal were able to sense negative energy and sadness from the parents. Mothers stayed strong to who they were, as a way to demonstrate to their gaaynggal their strength but also their strength as Aboriginal people. *“I try not to be sad because my babies can feel me and I don’t like it”* (Mother 10—Kamilaroi). 

This also included the strength it took to be away from their other gaayli; several of the parents had one or more gaayli being looked after by family, unable to visit the hospital or be with their parents.

**Theme (3) Covert racism and bias impacts culturally safe experiences**

*“Show more respect towards us, towards Aboriginals. They look at Aboriginal people and they judge them for their colour. And a lot of Aboriginal people are shy, they don’t like talking. And they think that Aboriginals don’t know what they’re doing, they think… They just think they’ve got authority over us, and they class us as second-class citizens.”*

(Mother 7—Gamilaroi)

Several parents reported experiencing racism that created mistrust in health professionals. Experiences of racist behaviour left parents on edge and unable to be themselves, fearing their gaaynggal might be labelled as at ‘*high risk’* for child protection, affecting their ability to see and care for their gaaynggal. As a result, families perceived the NICU environment as culturally unsafe and reported that they struggled to engage openly with the staff and services. After experiences of unfair treatment, parents felt that interactions with staff were more difficult, and it was harder to be open to a trusting relationship. *“Once you feel like that, they’ve treated you differently, you just don’t feel the same anymore. You just feel like, oh should I even be, like you’re a bit scared to even ask a question about things.”* (Mother 2—Aniwan).

Racism was not always reported as overt. Many parents reported experiencing covert racism, particularly through non-verbal cues such as staff facing their backs to parents when talking about the gaaynggal or avoiding eye contact when communicating with the family.

Parents who had short stays with full-term gaaynggal and parents who had long stays with extremely premature gaaynggal reported the same experiences. *“I see the body language first. If I can see you’re not going to treat me right, then I know it. I always read their body language, all the time.”* (Mother 10—Kamilaroi).

While less frequent, overt racism included comments regarding the parent’s skin colour, questioning about who was caring or responsible for their older gaayli, and comments about personal behaviour and physical presence in the NICU environment. *“I did say often, you’re treating me unfairly, she’s like, do you mean that in a racist way?… I said, no I do feel like I’m being treated fairly. And they’re like, oh well because of your skin colour or whatnot.”* (Mother 2—Aniwan).

Through sharing *concerns* about the racism experienced by parents, some mothers also reported concerns about child welfare involvement influenced by racial bias. Mothers reported feeling marginalized and targeted by health professionals. As one mother who had been in the NICU for 22 days described, *“I just get them thoughts that I come back up and my babies are not going to be here…Just being in the hospital, I feel like they’re watching me. I got all these thoughts that they’re going to take my babies away from me… ”* (Mother 10—Kamilaroi).

Racial bias in the hospital was reported by several parents, which affected their experience of culturally safe care in the NICU. This included a mother being offered nicotine replacement therapy, despite not being a smoker. “*They asked me did I need a patch. … I said what kind of patch? What do I need? They said, you need a patch to… So you can stop smoking. I said, look, I don’t even smoke cigarettes.”* (Mother 7—Gamilaroi).

These experiences were reported to differ from the observations of non-Aboriginal patients and their families. “*And I see that other nationalities get treated better than Aboriginals….”* (Mother 7—Gamilaroi).

**Theme (4) Health provider communication can obstruct parents’ experience of cultural safety**

Communication had the ability to create a positive experience for parents and reduce some of the harms of the NICU experience. Several parents reported regular and consistent positive communication with staff that was able to provide the level of insight and connection they desired. This was reported by parents who had gaaynggal who had been admitted to the intensive care environment. Fewer reports came from parents who had only experienced the special care nursery. *“His other nurse, the next day… She was so lovely. She let me have a cuddle with him. She said, don’t worry about the whys, and we can make it work. That’s the type of positive attitude I feel like they should have when they’re in the NICU. She was always explaining everything. She explained everything to me, and it was really helpful.”* (Mother 13—Wonnarua).

However, the parents emphasized that communication was also a key factor that could negatively impact their experience in the NICU environment and create a culturally unsafe space. All the parents reported experiencing poor communication, which caused barriers before, during, and after the NICU admission. “*… it’s like they’re communicating with the Aboriginals fellas that’s not really good. You see them communicating with each other and that’s perfect, why can’t they do that with us?”* (Mother 2—Aniwan).

Parents reported receiving second-hand information regarding their child from their partner or others; however, they felt that it would have been more appropriate to receive it directly from health professionals. This included clearer communication on being discharged from their service; there was a lack of support regarding transitioning to home and the baby’s needs post-discharge. *“We just went straight home, and I had a follow-up call to ask how my experience was, and I was honest with them, and just said communication was not there […] How’s her feeding going? How’s her jaundice looking? That would’ve been nice. ”* (Mother 4—Worimi).

There was significant concern caused by the inability to ‘*consent*’ to medical care and care plans for their gaaynggal. Some parents described feeling that staff withdrew information from them because they were “young” parents.

**Theme (5) Recommendations to uphold culturally safe care in NICU**

The NICU environment was reported to impede cultural care and support that was inclusive of family and community due to the nature of and the resources required in the environment. Parents recommended spaces to facilitate family and community visiting the parents and baby as an area for change to uphold culturally safe care.

Many mothers recommended establishing an Aboriginal and Torres Strait Islander support group for parents whilst their gaaynggal were admitted to the NICU to facilitate connection and support for each other through their journey. The parents who were able to connect with other Aboriginal families in the NICU reported that it was beneficial to their own journey and recommended placing Aboriginal gaaynggal in close proximity to a support connection. “*Because you get to talk to other Indigenous families and stuff. …You create that bit of a bond because obviously you would with anybody else but I think you just tend to stick closer to those other Indigenous families because you understand that this is not where we’d usually have a baby.”* (Mother 15—Gamilaroi).

A few parents described the inclusion of cultural mementos, such as Aboriginal cot cards, humidity crib covers, and breastfeeding cuddle hearts, during their NICU admission as being a positive inclusion. *“I feel like adding all of the culture stuff to the baby’s crib is important. It takes them back to home and back to their roots.”* (Mother 13—Wonnarua).

All the parents reported at least one interaction with an Aboriginal liaison officer (ALO) or Aboriginal staff member and the positive impact it had on their experience. The ALO for the children’s hospital was praised by many of the parents as an integral part of the multidisciplinary team, and the parents recommended that there should be more Aboriginal staff of all levels of expertise; they should be employed in the NICU environment to make the experience more culturally safe as they would apply an Indigenous lens to the care provided. *“They should have more Aboriginal workers up here. … I reckon they need more Aboriginal social workers in the hospital… Even more Aboriginal nurses that actually knows what Aboriginal people go through.”* (Mother 10—Kamilaroi).

Additional quotations supporting these findings are presented in Table 2.

## 4. Discussion

This study found that an admission to a NICU can disrupt the journey from birthing to parenthood and impact culturally safe caring practices for Aboriginal parents and their gaaynggal. This paper aimed to privilege Aboriginal parents’ voices and explore their experiences of the NICU to inform culturally safe care environments.

*“So a lot of things need to be done. The whole hospital system with Aboriginals, need to be changed.”*

(Mother 7–Gamilaroi)

The environment and the nature of the NICU were found to cause trauma, as well as trigger trauma, for Aboriginal parents. Experiences of separation and the lack of a parental role in the NICU are is commonly known in the non-Indigenous literature to impact and disrupt bonding and attachment in the parent–child relationship [28]. It is also known that trauma can be triggered in the transition to parenthood, impacting the parent–child relationship [29]. For Aboriginal parents, the NICU can amplify that trauma and impede the capacity to nurture and care for their gaaynggal. This is due to the ongoing historical and contemporary effects of colonisation inflicted on Aboriginal people in Australia, particularly during the assimilation policy, when Aboriginal gaayli were removed from parents based on race and stripped of their cultural identity [30,31]; this has created stolen generations of Aboriginal *gaayli* and the breakdown of parenting roles, and there has been growing evidence of impacts on health and wellbeing [30,31]. Presently, there continues to be an over-representation of Aboriginal gaayli in out-of-home care; they are 5.6 times more likely to be reported to child protection and 10.8 times more likely to be removed than a non-Indigenous child [32]. In this study, the separation of mother and gaaynggal caused fear and anxiety of their gaaynggal being taken, due to family history of forced removal (stolen generations) and current (out-of-home care) statistics. It also impacted the ability to uphold cultural caring practices for their gaaynggal; the parents in this study reported a lack of ability to be involved and to include family and kinship support in their care and that they spent time away from country due to the nature of the NICU setting. This coincides with research across all Aboriginal health spaces, which details the impact this separation can cause to the health and wellbeing of Aboriginal people [30].

These findings coincide with other Indigenous-led evidence that reported parents’ concerns regarding Indigenous child removal in the NICU, the inability to parent ‘our’ way [13], and the compounding effects that can be triggered by intergenerational trauma cycles when exposed to the NICU environment [12,13]. It was highlighted in First Nation Canadian NICU research that when the physical, developmental, and emotional needs of the parents are met culturally, it will enable a holistic care approach that is culturally safe in nature [13]. The principles of a trauma-informed care have been recommended internationally to be implemented in the NICU settings to increase culturally safe care [13]. In Australia, trauma-informed frameworks have been co-designed with Aboriginal and Torres Strait Islander communities for parents who experience complex trauma in the perinatal period [33], and there have been recommendations on safe strategies and partnerships that work using an Aboriginal lens [34]. These principles have been recommended to be embedded in maternity services [35]. The findings from this study indicate a need to extend beyond the maternity sector and to be embedded into the NICU setting.

Aboriginal parents in our study reported experiencing racism and cultural bias during their NICU experience. Racism, discrimination, and unfair treatment in public health services such as hospitals have been reported to persist and shape inequitable healthcare and to cause barriers to access [36]. These findings align with recent research undertaken with Aboriginal and Torres Strait Islander mothers in maternity care in South Australia, which saw mothers experience stress and a lack of personal control due to racism during their care [37]. Indigenous-led evidence has reported that Aboriginal and Torres Strait Islander people who experience episodes of everyday racial discrimination are three times more likely to have high or very high psychological distress compared to those who reported no episodes [38]. It is recommended that a whole-system approach to antiracism be undertaken in hospital settings [38]. The findings of this study supported Aboriginal research on racism and indicate the importance of the need for antiracism guidelines and/or policies in neonatal settings to support a culturally safe and responsive environment for Aboriginal parents to thrive in during their NICU admission.

This study found that health care provider communication could obstruct the parents’ ability to experience culturally safe care in the NICU. Although cultural mementos were appreciated by the parents and included into their gaaynggal’s care, parents recommended deeper culturally safe practices that would support them through their NICU journey; this included communication. It is well established that effective health provider communication is critical in effectively supporting parents in the neonatal setting [39] and requires four functions (building and maintaining relationships, exchanging information, sharing decision making, and enabling parent self-management) to be successful [40]. This evidence has informed the development of a NICU communication framework. Findings from this study and other Indigenous-led explorations of NICU have recommended further education to build the confidence of health professionals and clear guidelines on implementing effective communication strategies [11]. To improve the quality of culturally safe care for Aboriginal parents and the gaaynggal’s experiences in the NICU, more research is required to embed guidelines into clinical practice.

### Strengths and Limitations

The study findings should be interpreted with acknowledgement of the following study strengths and limitations. One clear strength of this study is that it was conducted by an Aboriginal mother and neonatal nurse, which aligns with Indigenous methodologies that emphasize relationality as a key component of successful research with Aboriginal people. This perspective is essential in ensuring that the voices of Aboriginal parents are prioritized. However, there are potential limitations to this approach as it also has the potential to introduce bias due to the researcher’s personal experiences and background. Another limitation is that the study was conducted at a single site in New South Wales, Australia, which may not fully represent the diverse experiences of Aboriginal families across different regions. As such, the findings should be interpreted with an understanding of this geographical and cultural diversity. A final limitation is that during the recruitment period, no Torres Strait Islander gaaynggal were admitted to the NICU, and therefore, their experiences are yet to be privileged.

## 5. Conclusions

The Aboriginal parents reported experiencing trauma and racism in the NICU environment, which are compounded by barriers to the upholding of cultural care practices and communication with healthcare providers. Culturally safe care practices have been recognised as critical to support Aboriginal parents through the neonatal journey [4]. In collecting stories, this study has identified key areas for improvement to create and embed culturally safe care in the NICU. Through further cultural safety education and training and the facilitation of Aboriginal family connections and support groups and culturally inclusive spaces to support parents and their kinship systems, the NICU environment can begin to meet the cultural care needs of Aboriginal parents and their gaaynggal during their admission. To both further improve culturally safe care and facilitate cultural care practices for Aboriginal parents, it is highly recommended that Aboriginal staff representations be increased across all levels of health professional experience, including within clinical coordinating roles. These recommendations are voiced by Aboriginal parents as ways to provide culturally safe care in the NICU in this local setting. To further build on culturally safe caring practices in the NICU for Aboriginal parents and their gaaynggal, further research is suggested at local and national levels.

## Figures and Tables

**Table 1:** Aboriginal gaaynggal demographics.

Parent/s	Aboriginal and/or Torres Strait Islander Identity	Gestation Age of Infant at Birth in Weeks and Days.	Infant Age at Interview	Type of Care Received	Geographical Location *	Connection to Country
Mother 1	Aboriginal	40.4	2 days	Special care	Major City	Awabakal
Mother 2 and Father 3	Aboriginal	36.6	4 days old	Special care	Inner Regional	Aniwan
Mother 4	Aboriginal	39 + 1	29 days old—discharged	Special care	Inner Regional	Worimi
Mother 5	Aboriginal	34 + 5	4 days old	Special care	Major City	Wonnarua
Mother 6	Aboriginal	38 + 2	14 days old	Intensive care	Outer Regional	Kamilaroi/Gamilaroi
Mother 7 and Father 8	Aboriginal	27 + 5	6 weeks old	Special care and Intensive care	Inner Regional	Kamilaroi/Gamilaroi
Mother 9	Aboriginal	27.5	9 weeks old	Special care and Intensive care	Inner Regional	Kamilaroi/Gamilaroi
Mother 10 and Father 11 *	Aboriginal	33.2	22 days old	Special care and Intensive care	Outer Regional	Kamilaroi/Gamilaroi
Mother 12	Aboriginal	28.1	8 weeks old	Special care and Intensive care	Inner Regional	Birripi
Mother 13	Aboriginal	32.0	6 weeks old—discharged	Special care and Intensive care	Major City	Wonnarua
Mother 14	Aboriginal	26.3	7 weeks old	Special care and Intensive care	Inner Regional	Birripi
Mother 15	Aboriginal	26.4	13 weeks old	Intensive care	Inner Regional	Kamilaroi/Gamilaroi

* Australian Statistical Geography Standard (ASGS) Edition 3 [24].

**Table 2:** Additional theme quotations.

Theme/Subtheme	Illustrative Quote
**Theme 1:** Trauma and its triggers in the NICU	“When he was born, chucked on my chest straightaway, taken off straightaway, checked over quickly and then he got taken straight to NICU” (Mother 1—Awabakal). “Yes, to this day I still cry about that. I’m allowed to go and pick him up whenever I like, but if that just pops in my mind, I sit there and have a cry. I’m like, I still don’t understand why I couldn’t physically be with him after all that. So yes, it still makes me cry every now and then.” (Mother 9—Gamilaroi) “That was scary within itself, but then in the first few hours I was like, I didn’t just have a child. I refused to believe that I’d just had a child. So then it took a couple of days to actually comprehend that. Then we came into NICU, and being separated because I had to go into a different ward, being separated was hard” (Mother 9—Gamilaroi). “I was sitting there, will she ever be able to breathe without a ventilator? Will she be able to live a somewhat normal life where she doesn’t have to have something hooked up to her to be able to help her breathe?” (Mother 15—Gamilaroi). “We just want to get out, we just want to walk out but we can’t, we’ve got to be here for this little baby, it’s hard. I’ve said that many times. We have breakdowns, but we pick ourselves back…But we’re here, we’re always here, and we’ll always be here, that’s our baby.” (Mother 7—Gamilaroi). “It was very hard; it took a lot out of us. Feels like we’re still recovering from how much actually drained us, we’re still, I don’t know how to really explain it… I feel that, up and down, it’s so touch and go, you don’t know if it’s good or bad or whatever. And the way they explain things too is very bland, they keep a straight face, they don’t show no emotion. Even when we’re upset, they just still keep it, they don’t even… You could just pat us on the back or whatnot, reassure us he’s going to be fine, they don’t really do that. They just leave us wondering and we go back to where we’re staying, and we just get upset and cry and panic.” (Mother 2—Aniwan). “Especially from most Aboriginal communities, they’re remote or inland. Where this is a city, so I’ve never seen so many buses in my life for the same number. It is ridiculous. Whereas we only have five buses back where we’re from and they go to specific places. Whereas this place is few, few, few. And yes, it’s bigger. Bigger area, bigger town. You don’t know anyone really. Unless you know people from where you’re going to. I don’t know anyone.” (Mother 15—Gamilaroi)
**Theme 2:** Aboriginal cultural caring practices are not upheld in the NICU	“…just pissing me off because, like I said, they want to interact when I’m in there with them. Let me be with my babies, you’re not letting me be with them.” (Mother 10—Kamilaroi) “I had a nurse give him a bottle without my consent.” (Mother 13—Wonnarua) “I should be the fucking first person in on that plan, straight away. Sometimes I feel like swearing and going off with them, but I really can’t. Not when my babies are in this position at the moment.” (Mother 10—Kamilaroi). “Yes, that strength just got pulled out of nowhere. And I feel like I can do anything with her by my side. It’s scary and it is a rollercoaster, two steps forward, ten back.” (Mother 12—Biripi) “We know every little detail. And when we do ask questions, they’re like, ah you’re asking this again, or whatnot. I want to be reassured 20 times a day if I can be, I don’t really care. I would rather be told that he’s fine heaps of times, every time I ask.” (Mother 2—Aniwan). “It’s hard enough you have to leave your baby here and go home, or whatever. And then you come back here, and you can’t really talk to your baby or do this for your baby or do that for your baby. It’s frustrating.” (Mother 13—Wonnarua). “Yesterday when he was underneath the light the whole time, I did not hold him all day. The whole entire time I was here I didn’t hold him until today. It just hurts me when they do stuff and they’re not doing it properly.” (Mother 5—Wonnarua). “When I do go in there, my babies, when they’re hungry, it’s like they don’t like me… When I want to breastfeed, they say you’ve got to wait. I’ve got to wait until their feeding time. I do understand that I need to wait for their feeding time, but if they’re fucking hungry, I’m going to give them a feed. They are not starving another 20 min until when I’ve got the milk they need.” (Mother 10—Kamilaroi).
**Theme 3:** Covert racism and biases impact culturally safe experiences	“We don’t need that, tolerating us.” (Father 8—Gamilaroi) “They are just watching him all the time, and asking him does he know what he’s doing, and… He’s a father of seven kids.” (Mother 7—Gamilaroi) “I was sitting there giving eye contact and they still wasn’t looking at me.” (Father 3—Gamilaroi). “I just feel like, some of them, I feel like that as soon as you go out of the room that they talk about you and stuff…They’re racist… they don’t see where we’re coming from.” (Mother 5—Wonnarua) “You get this feel of them staring at you? They were just all staring, and all having a little whisper. And that’s why I was thinking, and what are they are all going on about.” (Mother 7—Gamilaroi) “I see the body language first. If I can see you’re not going to treat me right, then I know it. I always read their body language, all the time. There’s that one nurse in there…but she always tries to help me all the time. If I’m doing something, like if I’ve got babies, she’s always got to say something, like if I’m doing it wrong. But I’m not even doing it wrong.” (Mother 10—Kamilaroi). “I try to tell them, as well. Is it okay for me not to come up here? But I couldn’t because I know, like I said, I can see the racism between my babies because they’re black.” (Mother 10—Kamilaroi). “We would’ve liked more information stuff, it’s like they didn’t want to give us, but every time we… I did say often, you’re treating me unfairly, she’s like, do you mean that in a racist way?… But a couple of the workers that come into me said, what are you feeling about, because I said, no I don’t feel like I’m being treated fairly. And they’re like, oh well because of your skin colour or whatnot, and stuff like that.” (Mother 2—Aniwan). “While we’re waiting for the doctor, she came in with a piece of paper and she said, so I want to know your name of your children, and date of births, and who’s got your children.” (Mother 7—Gamilaroi). “Just being in the hospital, I feel like they’re watching me. I got all these thoughts that they’re going to take my babies away from me and I don’t like it. “(Mother 10—Kamilaroi). “Constantly watching us. When we come for baby, constantly watching us. And we could be just sitting there with baby, holding baby, talking amongst ourselves. And they’re making out… They’re coming up, fiddling around. And just the… And if we wanted their attention, it’ll take forever for them to come to us.” (Mother 7—Gamilaroi).
**Theme 4:** Health provider communication can obstruct parents’ experience of cultural safety	“I rang up straight away, and I said, why aren’t I getting updates? And then I was told that they thought my partner had told me everything. He had told me some things, but he’s not there, he’s not a doctor, he’s not a nurse, he can’t translate it the way they can.” (Mother 1—Awabakal). “They said I could have a cuddle, and then it didn’t turn out that I could have a cuddle, and she was heartless about it. It was confronting for me, because he was only born 12 h before, and I hadn’t even held him. She wasn’t sympathetic about it. She was very blunt and instead of explaining why, she just said no. And I was like, okay. I went back to my room, and I was disheartened because I was like, I’m never going to get to hold him.” (Mother 13—Wonnarura). “I think that the nurses need to explain more for starters, even though it’s in the beginning and it’s hard for you to take it in, but I don’t remember most of it for about two weeks. But even just that, having it explained multiple times even, because I stood there for days looking at him like, what’s wrong with him? Didn’t get told that he couldn’t breathe by himself. I didn’t realize that. So they’re like, oh yes, this is him.” (Mother 9—Gamilaroi). “That’s like being a young mum, and I know how easy it is for them to just override me, and us young parents, but yes, it’s not my first time. It was my second time. I know when something’s up, so yes, it’s just really hard when they don’t listen.” (Mother 4—Worimi). “I would’ve liked to have been told why [feeding tube], and a little bit more information would’ve been good. And before they did it, it would’ve been nice to be like, oh, yes, we’re going down this path because she’s not feeding well, blah, blah, or whatever the reason was. So, yes, I was pretty upset at the fact that I couldn’t just receive a quick call of why. And it was harder for me because I wasn’t physically allowed up there, so that was really, really hard. And then just, I don’t know, just seeing my partner up there, like he would Facetime me and stuff, but it’s obviously different.” (Mother 4—Worimi). “I thought they had to ask for consent to get the feeding tube in, and all that kind of stuff, so it was a bit confronting seeing her with that, when I didn’t know that she needed to get that.” (Mother 1—Awabakal). “I had to make that apparent to most nurses. I had to tell them I wasn’t told this information so anything that happens, please tell me. I wasn’t sure. I thought she maybe not have told me because a medical team, she thought maybe the medical team was going to come around and talk to me. But I had no one talk to me.” (Mother 15—Gamilaroi). “One of the specialists said to me when I was only just watching what they were doing. They were like, he asked me to move back or whatnot, and can you ask questions after we’re finished doing, not while we’re doing it. And they only just sitting there putting drips in, I just wanted to ask what are you doing there, nothing terrible, but they just the fact that he was like, can you wait till after we finish, that’s a bit…I’m only just, I’m not in your way, I’m not touching nothing or anything, just talk me through it while you’re doing it, or if not, just I don’t know. It was just seemed a bit rude.” (Mother 2—Aniwan). “Once you feel like that, they’ve treated you differently, you just don’t feel the same anymore. You just feel like, oh should I even be, like you’re a bit scared to even ask a question about things.” (Mother 2—Aniwan).
**Theme 5:** Recommendations to uphold culturally safe care in NICU	“It’s like there’s not enough support for Aboriginal people isn’t it, it’s good.” (Mother 2—Aniwan) “And being away from the other kids… They ring up all the time and want to come down here… especially that they weren’t allowed to see her in that room, in the special care room in… They don’t allow kids in there, to go and see the babies. Yes, so they couldn’t see her, but they’re allowed to come and see her here, but I’ll just leave them at home for now.” (Mother 6—Kamilaroi). “I feel like adding all of the culture stuff to the baby’s crib is important. It takes them back to home and back to their roots.” (Mother 13—Wonnarua). “The really good support that we had was with the Aboriginal workers here, but it’s not anything bad or anything, we just feel comfortable speaking to someone that understands us.” (Mother 2—Aniwan). “Once we got linked in with the ALO and stuff like that, that was good. She got to step in and help him there for a little bit until he had to go up to the hospital here. Yes, that was good. He really liked it and she was amazing… I think obviously having Aboriginal liaison officers are really good because you’re able to talk to someone who understands what you need culturally as well.” (Mother 15—Gamilaroi). “Aboriginal staff know culturally that’s the norm for most Aboriginal people to have those certain experiences throughout their life.” (Mother 15—Gamilaroi). “They should have more Aboriginal workers up here. Especially just walking around, checking on them. Walking around, seeing if everybody’s okay. Because the nurses, they don’t actually know if you’re okay or what are you going through. They’re not going to have a yarn to you, they should. I reckon they need more Aboriginal social workers in the hospital… Even more Aboriginal nurses that actually knows what Aboriginal people go through. Especially their background, as well, they should understand.” (Mother 10—Kamilaroi). “It’s hard just not having the support people, either. That’s probably the hardest bit. Just to have people around. I know they probably can’t hold him either, but just to have someone with me.” (Mother 1—Awabakal) “I reckon for Aboriginal dads. There could be something. And obviously for Aboriginal mums but I think more for Aboriginal dads because I don’t think, obviously men struggle in the Aboriginal community as much as it is. So, I think them having a kid and stuff here is obviously a lot different for them too and what they know.” (Mother 15—Gamilaroi). “Having something where other Indigenous mums, they get together and they’re able to, like we do…So, I think that is nice to know that there are other families or there’s someone out there you probably know but you haven’t met them before, but they know your family, you know theirs. And you just haven’t crossed paths yet.” (Mother 15—Gamilaroi). “That was so awesome. Having [parent name], who was going through a similar… She had twins and only one of her twins was admitted to the NICU and her twin had problems with her heart. She was on the same journey. The up and down, the side to side, and it was just really nice to have her there and she made it so much more bearable. And we cried together, and we would always talk together, and she would let me hold her other twin, the one that wasn’t in the NICU, and it was really nice to have someone who gets it. And she wasn’t the only friend I made. There were a few in there that we had exchanged numbers and Facebook, and they were really helpful.” (Mother 13—Wonnarura). “Especially for the ones that do come off country, like [mother], she was an Aboriginal mum, I’m pretty sure she was a first-time mom. She had twins and she was in there for the same, well longer than I was, but she went home before I did. And she was very inspiring because she, being a first-time mom, she used to tell them off, and tell them what to do, and, no, you’ve got to call me before you just take blood from my baby. And I feel like that was inspiring, and she used to call them out on their shit, and I feel like that was good.” (Mother 13—Wonnarura).

## Data Availability

In line with Indigenous Data Sovereignty and Aboriginal and Torres Strait Islander Ethical Research Principles, no data sharing is available from this study.

## Abbreviations

The following abbreviations are used in this manuscript:

NICU	Neonatal Intensive Care Unit
JHCH	John Hunter Children’s Hospital
ALO	Aboriginal Liaison Officer
